# Disc‐like lesions in the intestinal tract after renal transplantation

**DOI:** 10.1002/ccr3.5645

**Published:** 2022-04-12

**Authors:** Arjan J. Kwakernaak, Rob W. van der Pluijm, Neelke C. van der Weerd, Lianne Koens, Wytske M. Westra, Krisztina B. Gecse, Ankie Kleinjan

**Affiliations:** ^1^ Department of Internal Medicine Division of Nephrology Department of Rheumatology and Clinical Immunology Amsterdam University Medical Centers, University of Amsterdam, Amsterdam Rheumatology and Immunology Center Amsterdam The Netherlands; ^2^ Department of Internal Medicine Division of Infectious Diseases Amsterdam University Medical Centers, University of Amsterdam Amsterdam The Netherlands; ^3^ Department of Internal Medicine Division of Nephrology Amsterdam University Medical Centers, University of Amsterdam Amsterdam The Netherlands; ^4^ Department of Pathology Amsterdam University Medical Centers, University of Amsterdam Amsterdam The Netherlands; ^5^ Department of Gastroenterology and Hepatology Amsterdam University Medical Centers, University of Amsterdam Amsterdam The Netherlands; ^6^ Department of Internal Medicine Division of Haematology Rivierenland Hospital Tiel The Netherlands

**Keywords:** EBV, immunosuppression, lymphoma, PTLD, renal transplantation

## Abstract

We report a case of intestinal lesions in a patient with a history of lupus nephritis and renal transplantation. Biopsy revealed an EBV‐driven post‐transplant lymphoproliferative disease (PTLD). An EBV‐driven PTLD is a major complication after renal transplantation and is an important differential diagnostic consideration in the follow‐up of renal transplant recipients.

## Graphical abstract

Post‐transplantation lymphoproliferative disease (PTLD) is an important diagnostic consideration in a transplant recipient that presents with intestinal disc‐like lesions.

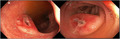

## 1 CASE DESCRIPTION

**FIGURE 1:**
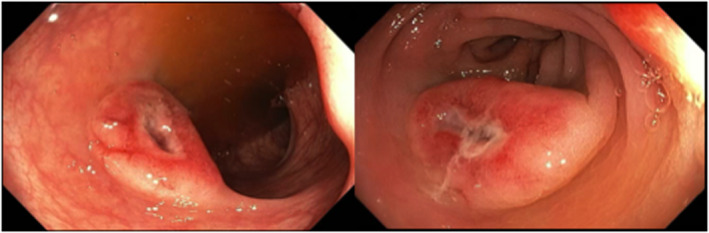
Disc‐like lesions with central ulceration found with endoscopy

A 33‐year‐old female patient was admitted because of abdominal pain and diarrhea. Her medical history was remarkable for lupus nephritis for which she had undergone a renal transplantation that was complicated by a severe rejection and treated with methylprednisolone, plasmapheresis, intravenous immunoglobulin, and antithymocyte globulin (ATG). Colonoscopy showed several disc‐like lesions with central ulceration in the colon as depicted in Figure 1.

## 2 WHAT IS YOUR DIAGNOSIS?

### 2.1 Discussion

**FIGURE 2:**
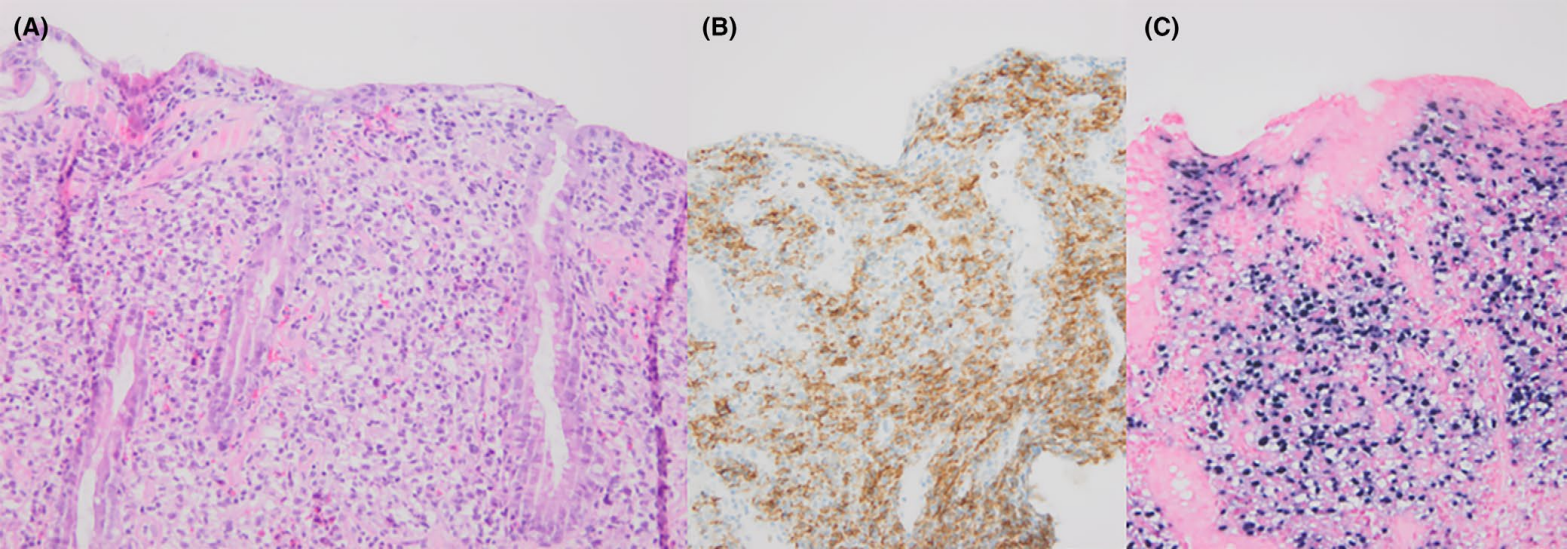
(A) HE section of the intestinal mucosa, showing large, atypical lymphocytes between the crypts. (B) The large lymphocytes show positivity for B‐cell marker CD20. (C) There is also extensive positivity in the EBER assay

Light microscopy of the biopsy specimens demonstrated infiltration of large lymphoid cells (Figure 2) that were positive for CD20, CD79a, CD30, Mum‐1, BCL2, and c‐Myc on immunohistochemical staining. Clonality analysis confirmed the presence of a monoclonal B‐cell population. Polymerase chain reaction (PCR) detected viral ribonucleic acid (RNA) of Epstein–Barr virus (EBV) and EBER (in situ hybridization for EBV‐encoded RNA in infected cells) was positive. Of note, both kidney donor and recipient were EBV IgG‐positive pre‐transplantation. We diagnosed her with EBV‐driven, monomorphic, post‐transplantation lymphoproliferative disease (PTLD). Her immunosuppressive regimen was reduced and she started with rituximab that resulted in complete remission.

Post‐transplantation lymphoproliferative disease is a serious complication of organ transplantation with a spectrum ranging from clinical indolent to aggressive lymphomas.^1^ Well‐established factor for PTLD is type and cumulative amount of immunosuppression, in particular, T‐cell depleting therapy such as ATG.^1^, ^2^

**Learning points**

- Post‐transplantation lymphoproliferative disease is a serious complication of renal transplantation.
- Post‐transplantation lymphoproliferative disease is an important differential consideration in a transplant recipient that presents with intestinal disc‐like lesions.
- Established risk factor for PTLD is cumulative amount of immunosuppression.

## CONFLICTS OF INTEREST

Not applicable.

## AUTHOR CONTRIBUTION

Arjan J. Kwakernaak has co‐written the manuscript. Rob W. van der Pluijm has co‐written the manuscript. Neelke C.van der Weerd was medical supervisor during admission of the patient, provided important intellectual content and reviewed the manuscript. Lianne Koens provided details regarding the pathology findings, provided important intellectual content, and reviewed the manuscript. Wytske M. Westra and Krisztina B. Gecse provided details regarding the endoscopy findings and reviewed the manuscript. Ankie Kleinjan provided important intellectual content and reviewed the manuscript.

## ETHICAL APPROVAL

We adhered to the ethics guidelines. Written informed consent was obtained from the patient to publish this report in accordance with the journal's patient consent policy.

## CONSENT

Written informed consent was obtained from the patient to publish this report in accordance with the journal's patient consent policy.

## Data Availability

Data sharing is not applicable to this article as no new data were created or analyzed in this study.
